# German longevity study reveals novel rare pro-longevity alleles clustering in mTOR signaling pathway

**DOI:** 10.1007/s11357-025-01640-7

**Published:** 2025-04-15

**Authors:** Daniel Kolbe, Janina Dose, Pasquale Putter, Malte Ziemann, Matthias Laudes, Regeneron Genetics Center, P. Eline Slagboom, Andre Franke, Joris Deelen, Almut Nebel

**Affiliations:** 1 Institute of Clinical Molecular Biology, Kiel University, Kiel, Germany; 2 Molecular Epidemiology, Department of Biomedical Data Sciences, Leiden University Medical Center, Leiden, The Netherlands; 3 Institute of Transfusion Medicine, University Hospital Schleswig-Holstein, Lübeck, Germany; 4 Institute for Diabetes and Clinical Metabolic Research, University Hospital Schleswig-Holstein, Kiel, Germany; 5 Regeneron Pharmaceuticals, Inc., Tarrytown, NY USA; 6 Max Planck Institute for Biology of Ageing, Cologne, Germany; 7 Cologne Excellence Cluster on Cellular Stress Responses in Ageing-Associated Diseases (CECAD), University of Cologne, Cologne, Germany

**Keywords:** Genetics, Human longevity, Exome-wide association analysis, Rare variants, MTOR signaling

## Abstract

In this study, we investigated the contribution of rare coding variants to human longevity by analyzing whole exome sequencing data from 1245 German long-lived individuals (LLI) and 4105 geographically matched younger controls. We identified novel exome-wide significant associations at both the single-variant and gene level, with a significant over-representation of genes involved in mechanistic target of rapamycin (mTOR) signaling. As such, three rare single variants in the mTOR-pathway genes *RPS6*,* FLCN*, and *SIK3* were enriched in LLI. Additionally, *RWDD1* emerged as a strong candidate gene for longevity, with LLI exhibiting a statistically significant burden of rare missense variants in this gene. Other associations involved *PRAC2*, *SLC16 A6*, *FOCAD*, *IHH*, *MESD*, *HOXA4*, and *DNAJB13*. Furthermore, we observed an enrichment of protein-truncating variants in the genes *ASXL1* and *TET2* amongst LLI, likely as a result of clonal haematopoiesis. The study emphasizes the role of rare variants in human longevity, particularly through mTOR signaling.

**Supplementary Information:**

The online version contains supplementary material available at 10.1007/s11357-025-01640-7.

## Introduction

Whether an individual achieves longevity, i.e. survives well beyond their life-expectancy, is influenced by a wide range of factors. Some aspects are within a person’s control, such as diet, exercise, alcohol consumption and smoking habits. However, research on long-lived families has shown that there is also a significant heritable component involved. Genetic variation is estimated to contribute between 10 and 30% to the phenotype and may be even higher in cases of extreme longevity [1–3]. Over the past few decades, substantial efforts have been made to unravel the genetic architecture of longevity [4]. A key focus has been the analysis of large cohorts of long-lived individuals (LLI), who are defined as the top percentile (1–10%) survivors of their respective birth cohorts and are characterized by a delayed onset of age-related diseases, making them convincing models to study longevity and healthy aging [5]. By comparing the genetics of LLI to those of younger, geographically matched controls, researchers have been able to identify genes, or rather variants within genes, that influence the likelihood of becoming long-lived. Surprisingly, the number of associations that have been confirmed across multiple studies and populations has been limited to only a few variants, such as those in *APOE* and *FOXO3*, that explain only a small fraction of the total heritability of longevity [6, 7]. The genomic association studies that detected these variants primarily focussed on common variation (i.e. variants with a minor allele frequency above 1%), leaving rare variants largely understudied. Investigating rare variants could therefore be a promising approach to potentially uncover parts of the missing genetic heritability, particularly since the vast majority of variants expected to have a functional effect are predicted to be rare [8].

## Materials and methods

### Study population

Our study population was comprised of 1265 unrelated German LLI and 4195 geographically matched younger controls. An individual was classified as long-lived if they survived past the 95th age-at-death percentile of their respective birth cohort. The mean age of the LLI cohort was 98.9 years (range 94–110 years) and included 602 centenarians (≥ 100 years). The female to male ratio was approximately 2.7:1 in the LLI (which at the time of recruitment was population representative, according to https://www.destatis.de/), and 1:1.4 in the controls. LLI were mainly identified through local registry offices throughout the whole of Germany. They received letters explaining the study and requesting consent to send a questionnaire and blood sampling kit. Participants were encouraged to consult their general practitioner for assistance with blood collection and completing the questionnaire. Eligibility required good health, physical activity, absence of major diseases, and mental fitness to complete a standard health questionnaire. Further details on the criteria can be found in a prior study [9]. The population representative younger controls (mean age at inclusion 50.5 years, range 18–83 years) were recruited as part of the FoCus cohort [10]. The project was approved by the Ethics Committee of the Medical Faculty of Kiel, and written consent is available from all participants.

### Whole exome sequencing procedure

DNA for whole exome sequencing (WES) was extracted from whole blood samples of each study participant. WES was carried out by the Regeneron Genetics Center. Details on the wet-lab procedure, such as sample preparation and exome capture, as well as initial processing of the raw sequencing data, can be found in a previous study [12]. Briefly, targeted exome capture was performed using a slightly modified version of IDT’s xGenv1 probe library and then sequenced using 75 bp paired-end reads on an Illumina NovaSeq 6000. The raw sequencing data underwent quality control using FastQC (https://www.bioinformatics.babraham.ac.uk/projects/fastqc/). FASTQ files were mapped to GRCh38 genome reference with BWA-mem [12]. The resulting Binary Alignment and Map (BAM) files were evaluated to identify and flag duplicate reads with the Picard tools (https://broadinstitute.github.io/picard/), which were then excluded from downstream analysis. The sample-specific BAM files were converted to fully lossless Compressed Reference-oriented Alignment Map (CRAM) format.

### Variant calling and quality control

DeepVariant v1.5.0 [13] (model type = WES) was used to create Genomic Variant Call Format (GVCF) files from CRAM files, calling only variants from exonic regions. The per sample GVCFs were then converted to per sample VCFs and subsequently merged into a multisample VCF using GLnexus v1.4.1 [14] and bcftools v0.1.16 [15]. Initially, a per individual quality control step was carried out which removed individuals with discordant sex information between genetically determined and self-reported sex, heterozygosity rates lower or greater than four standard deviations from the mean, and second-degree or closer relatedness amongst other samples (estimated using the identity-by-descent metric—keeping the sample with the lower missingness). Population structure was accounted for, removing any outliers (principal component analysis, PLINK 1.9 [16]), as described in a previous study [17]. Overall, individuals had a maximum missingness rate of 0.02 and a Ti/Tv ratio between 2.78 and 2.97. In a second step, all half-calls produced by DeepVariant were set to no-calls, and variants were removed if they had missingness rates greater than 0.05, deviated from Hardy–Weinberg equilibrium (*p*-value < 1 × 10^−6^) or had more than two alternative alleles. Furthermore, to reduce the number of somatic mutations caused by clonal haematopoiesis, variants were removed if their variant allele fraction (VAF) was either lower or greater than the 1st and 99th percentile of the VAF of all variants, respectively. In total, 1245 LLI and 4105 younger controls, and 848,689 variants were included in the analysis. A total of 794,022 of these variants were rare, i.e. minor allele frequency (MAF) < 0.01, which included 501,010 missense, 260,346 synonymous and 23,524 protein-truncating variants (PTVs). The variant calling and quality control pipeline is available at https://github.com/d-kolbe/Longevity-ExomeSeq-variant-calling-and-QC-pipeline.

### Single-variant and gene-based association analysis

The association analysis of the exonic variants was carried out primarily using the R package STAAR v0.9.7 [18, 19], a variant-set test for association using annotation information. Firstly, variants were annotated using the open-source R program FAVORannotator [20]. To this end, the VCF files were converted to Genomic Data Structure (GDS) files, which were then annotated to produce annotated GDS (aGDS) files. Annotations included a set of annotation principal components (aPCs), as well as three commonly used integrative scores: CADD, FATHMM-XF and LINSIGHT. The aPCS were calculated from functionally similar predictive scores, summarized into the following categories: Protein-Function, Conservation, Epigenetics-Active, Epigenetics-Repressed, Epigenetics-Transcription, Local-Nucleotide-Diversity, Transcription-Factor and Mappability. Following the annotation of variants, sample-specific genetic principal components (PCs, not to be confused with aPCs, which are variant-specific) and an ancestry-adjusted sparse genetic relatedness matrix (GRM) were calculated with FastSparseGRM (https://github.com/rounakdey/FastSparseGRM) for all individuals, using data from an Illumina Infinium Global Screening Array- 24 that has been previously published for our study cohort [17]. A null model was then fitted with the phenotype, sex and first ten genetic PCs of the samples. The single-variant analysis only included variants if they had a minor allele count above 5 (MAC > 5), given the lack of power and reliability of statistical methods to identify variants with very low allele counts. The gene-based association analysis was performed using STAAR-O, an optimized mixed version of the burden, sequence kernel association (SKAT) and aggregated Cauchy association (ACAT) tests, using only rare variants (MAF < 0.01). STAAR-O, like other gene-based tests, aggregates all rare variants in a mask (e.g., all missense variants in a gene) to calculate a single summary statistic for that mask. The test was performed for three different masks of variants per gene: synonymous, missense and protein-truncating. PTVs included stop-gain, frameshift deletions and insertions, stop-loss and splice-site variants. To prioritize variants that are more likely to be causal, STAAR-O incorporated the previously annotated aPCs and integrative scores as variant-weights (only in the case of missense and synonymous variant-masks). A gene-mask was excluded from the test if the minimum cumulative allele frequency was below 10 (cMAC < 10) and/or the total number of variants below 5 (#SNV < 5). Given the exploratory nature of our study, we chose to apply multiple testing correction according to the Benjamini–Hochberg method, with a false discovery rate (FDR) < 0.05, to achieve a balanced trade-off between discovery and false-positive rate [21]. The odds ratios for rare single variants, as reported in the text and Table 1, were calculated using a contingency table with a Haldane correction (adding 0.5 to each cell) [22]. Both the single-variant and gene-based analyses were additionally conducted as conditional tests, accounting for *APOE* genotype.

**Table 1:** Significant results from the single rare-variant analysis using WES data from a cohort of 1245 LLI and 4105 younger controls. Odds ratios (OR) were calculated using the Haldane correction [22]. CADD = Combined Annotation Dependent Depletion; MAF = minor allele frequency; HOM = homozygous carriers of minor allele; LLI = long-lived 804 individuals; YC = younger controls; M = male; F = female; Pos = genomic position based on GRCh 38; NFE = allele frequency in non-Finnish Europeans [37]; NFE_OR = odds ratio 805 based on NFE

Chr	Pos	Ref	Alt	Gene	rsID	Consequence	CADD	*P*-value	*P*-value (FDR-adjusted)	OR	MAF	HOM	MAF_LLI	MAF_YC	MAF_LLI_M	MAF_YC_M	MAF_LLI_F	MAF_YC_F	NFE	OR_NFE
9	19379580	G	A	*RPS6*	rs189871470	synonymous	22.4	1.7x10^−07^	6.4x10^−3^	16.5	0.07%	0	0.28%	0.01%	0.75%	0.02%	0.11%	-	0.04%	6.49
17	48724360	T	TG	*PRAC2*	rs199607933	frameshift insertion	13.98	8.1x10^−07^	0.025	36.3	0.05%	0	0.20%	-	0.30%	-	0.16%	-	0.05%	4.19
17	68271344	G	C	*SLC16 A6*	rs112262188	synonymous	1.146	9.8x10^−07^	0.025	12.1	0.06%	0	0.20%	0.01%	0.60%	0.02%	0.06%	-	0.09%	2.36
11	116861300	A	G	*SIK3*	rs147730555	missense	19.45	1.6x10^−06^	0.034	43.0	0.06%	0	0.24%	-	0.45%	-	0.16%	-	0.01%	21.00
17	17215284	C	T	*FLCN*	rs41419545	missense	1.994	1.9x10^−06^	0.036	4.2	0.30%	0	0.72%	0.17%	1.20%	0.19%	0.55%	0.15%	0.32%	2.28
9	20770090	G	A	*FOCAD*	rs199678370	missense	20.5	2.3x10^−06^	0.036	9.9	0.05%	0	0.16%	0.01%	0.45%	-	0.06%	0.03%	0.01%	12.16
2	219055570	C	T	*IHH*	rs149554120	synonymous	14.13	2.4x10^−06^	0.036	3.3	0.15%	0	0.32%	0.10%	1.05%	0.17%	0.06%	-	0.27%	1.18

### Gene-set over-representation analysis

A gene-set over-representation analysis was performed using R package clusterProfiler [23] with function enrichGO for terms from Gene Ontology: Biological Processes (GO:BP) [24], as well as enrichKEGG for pathways from the Kyoto Encyclopedia of Genes and Genomes (KEGG) [25], for gene-sets between 20 and 200 genes. A pathway or ontology was only considered in our results if it contained at least two of our identified genes. A set of 18,492 protein-coding genes was used as background (universe). *P*-values were corrected for multiple testing by clusterProfiler, which accounts for redundancies within databases.

### Whole pathway analysis

A whole pathway analysis was performed in a similar way to the gene-based analysis, focusing on all pathways from KEGG. Given that STAAR-O is optimized for genes rather than gene-sets, we instead employed ACAT-V [26] which produced more conservative *p*-values, using the same three variant masks. *P*-values were corrected using an FDR < 0.05.

### Supporting evidence from other longevity cohorts

A look-up of rare coding variants that reached exome-wide significance in our dataset was performed in two distinct longevity cohorts.

The first consisted of 515 LLI and 496 younger controls of Ashkenazi Jewish (AJ) ancestry [27]. The AJ participants were recruited at the Albert Einstein College of Medicine since 1998 as part of the Longevity Genes Project (LGP) and the LonGenity study. AJ LLI were defined as individuals of age 95 years and above, with a mean age of 101 years. The younger controls were defined as individuals younger than 95 years without a history of parental longevity and had a mean of 83 years. The dates of birth of cases were confirmed by government-issued identification. The look-up of the summary statistics was performed at https://zdzlab.einsteinmed.edu/1/longevity.html [27].

The second cohort included 179 unrelated individuals selected from nonagenarian siblings from the Leiden Longevity Study [28]. The Leiden Longevity Study (LLS) is a family-based study that includes families with at least two full-blood nonagenarian siblings of Dutch descent (age at recruitment male ≥ 89 years, female ≥ 91 years). The siblings were enriched for familial longevity, defined as (1) having at least one parent who is amongst the top 10% survivors of his/her birth cohort and (2) at least two nonagenarian siblings in the same sibship who are also amongst the top 10% survivors of their birth cohort. The 179 cases had a mean age of 94 years (range 89–103 years). The whole-genome sequencing procedure is described in detail in a prior study [29]. Additionally, 98 unrelated controls from the Genome of the Netherlands Consortium (BBMRI-NL, age at inclusion < 65 years) were included [30]. Participants of BBMRI-NL are not selected for specific characteristics other than that they should reflect a random sample of the apparently healthy Dutch population.

### Software

Python version 3.9.4 [31] and R version 3.5.1 [32] were employed for data analysis. Nextflow 23.04.3 [33] was used in the processing of raw genetic data. Figures and graphics were generated with Seaborn v0.12, Plotly 5.8 [34, 35] and BioRender (https://www.biorender.com/).

## Results

### Rare variants in German longevity cohort

**Fig. 1:**
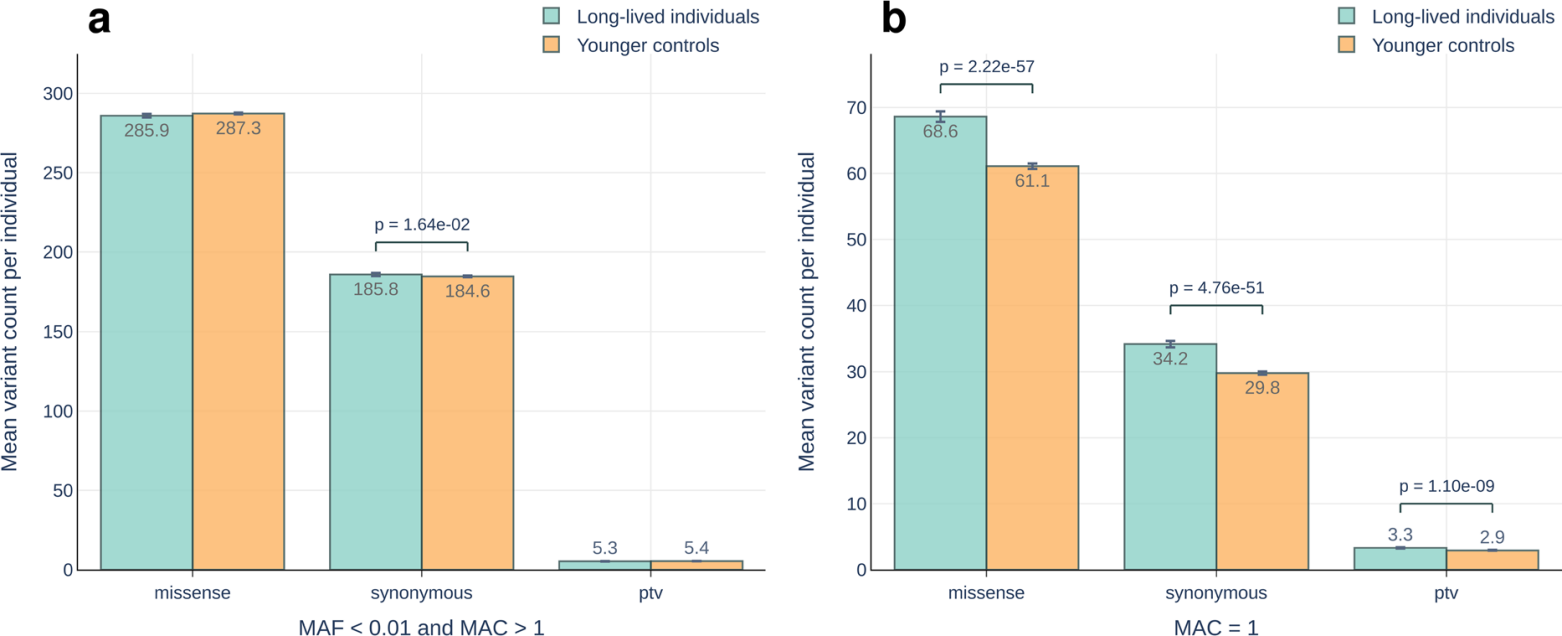
Mean count per individual of **a** rare alleles excluding singletons and **b** singletons only. *P*-values were calculated using a linear regression model fitted with sex and first ten genetic PCs

In this study, we performed a case–control association analysis focusing on rare coding variants in a German longevity cohort comprised of 1245 LLI (mean age at inclusion 98.9 years, range 94–110 years) and 4105 younger controls (mean age at inclusion 50.5 years, range 18–83 years). We observed that long-lived cases exhibited a higher number of mean rare alleles per individual compared to the younger controls. This difference resulted from an excess of singletons (i.e. variants present in a single individual in the cohort) in LLI, which was true for missense, synonymous and PTVs alike (Fig. 1), and to a small degree from an excess of non-singletons in the case of synonymous variants. There was no difference in the VAF of singletons in LLI compared to younger controls, indicating that the excess of singletons did not result from somatic mutations, such as those driving clonal haematopoiesis of indeterminate potential (CHIP) [36].

### Single-variant association analysis

**Fig. 2:**
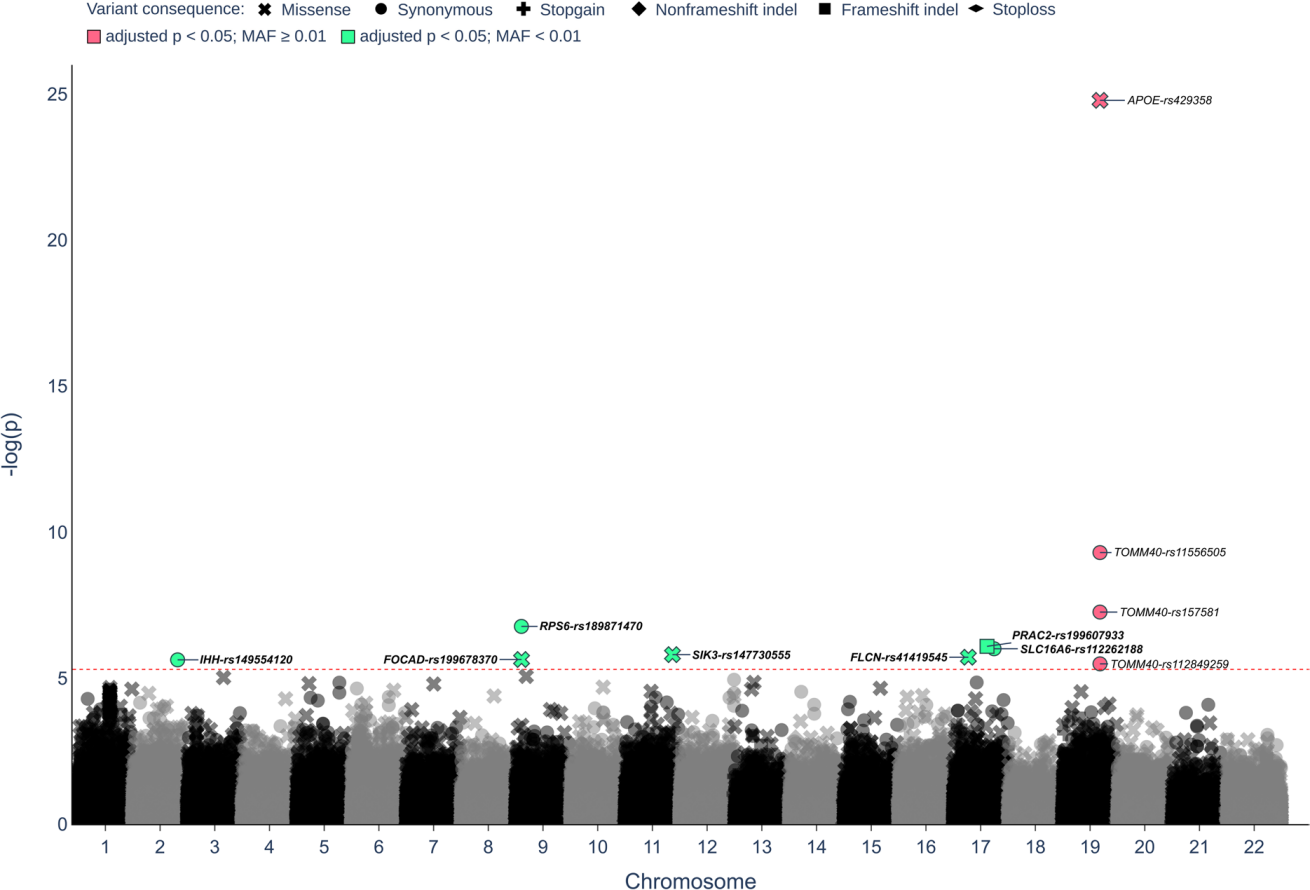
Exome-wide single-variant association analysis using WES data of 1245 German LLI and 4105 geographically matched younger controls. The association was performed using the R package STAAR-O [18], using sex and first ten genetic principal components as covariates. The significance threshold based on an FDR multiple testing correction of < 0.05 is indicated as a red dotted line. Variants that passed this threshold are labelled with *gene-rsID* and are colored (green = rare variants; red = common variants). Variants labelled in bold exhibited an enrichment of the minor allele in LLI (i.e. OR > 1)

We performed a single-variant association analysis of all protein-coding variants (Suppl. Data 1). Due to the lower reliability and statistical power of rare-variant analyses, we only included variants with a minor allele count greater than or equal to 5 (MAC ≥ 5). Ultimately, 54,406 common variants (MAF ≥ 0.01; 26,852 synonymous, 26,559 missense, 384 non-frameshift indels, 301 frameshift indels, 272 stop-gain and 38 stop-loss variants) and 97,993 rare variants (MAF < 0.01; 58,263 missense, 36,271 synonymous, 1169 frameshift indels, 1113 non-frameshift indels, 1111 stop-gain and 66 stop-loss variants) were analyzed. Amongst common variation, we observed that only variants in the known *TOMM40/APOE/APOC1* longevity hub achieved exome-wide significance (Fig. 2), with *APOE-rs429358 (ε4)* exhibiting the lowest *p*-value (*p* = 1.64 × 10^−25^, OR = 0.45). The other known longevity variant in *APOE*, *rs7412 (ε2)*, reached nominal significance (*p* = 5.4 × 10^−4^, OR = 1.34). The analysis of rare variants revealed seven novel exome-wide significant (FDR < 0.05) associations (Fig. 2): three missense variants—*SIK3-rs147730555-G* (adjusted *p*-value = 0.034, OR = 43.0), *FLCN-rs41419545-T* (adjusted *p*-value = 0.036, OR = 4.2) and *FOCAD-rs199678370-A* (adjusted *p*-value = 0.036, OR = 9.9); three synonymous variants—*RPS6-rs189871470-A* (adjusted *p*-value = 0.0064, OR = 16.5),* SLC16 A6-rs112262188-C* (adjusted *p*-value = 0.025, OR = 12.1) and *IHH-rs149554120-T* (adjusted *p*-value = 0.036, OR = 3.3); and one frameshift insertion—*PRAC2-rs199607933-TG* (adjusted *p*-value = 0.025, OR = 36.3) (Table 1). Conditioning the analysis on *APOE* genotype did not significantly impact the *p*-values of rare variants. In addition, we did not observe significant evidence of population structure (Suppl. Figure 1a). In all cases, the rare allele was present at higher frequencies in the LLI compared to younger controls, with two variants, *SIK3-rs147730555-G* and *PRAC2-rs199607933-TG*, being absent in controls altogether. Interestingly, the association signal appeared to be driven by male LLI, who had higher MAFs for all variants compared to their female counterparts. Nonetheless, the MAFs of all variants were higher in female LLI compared to younger female controls. Given that the size of our control group, whilst large for a genomic study, potentially lacks the sensitivity to detect all rare alleles in a population, we additionally compared the MAFs of LLI and younger controls to those of a large genomic dataset of ~ 580,000 non-Finnish Europeans (NFE – gnomAD v4.0.0) [37]. We observed that all rare variants had a lower MAF in NFE compared to LLI, with especially *SIK3-rs147730555-G* and *FOCAD-rs199678370-A* exhibiting high ORs. However, the OR for some alleles, such as *SLC16 A6-rs112262188-C* and *IHH-rs149554120-T*, were considerably lower than those calculated within our cohort. However, it is worth noting that there are significant population-level genetic differences between NFE and our study cohort, given that the large majority of NFE individuals are British (resulting from an integration of the UK Biobank into gnomAD v4.0.0).

### Gene-based association analysis

**Fig. 3:**
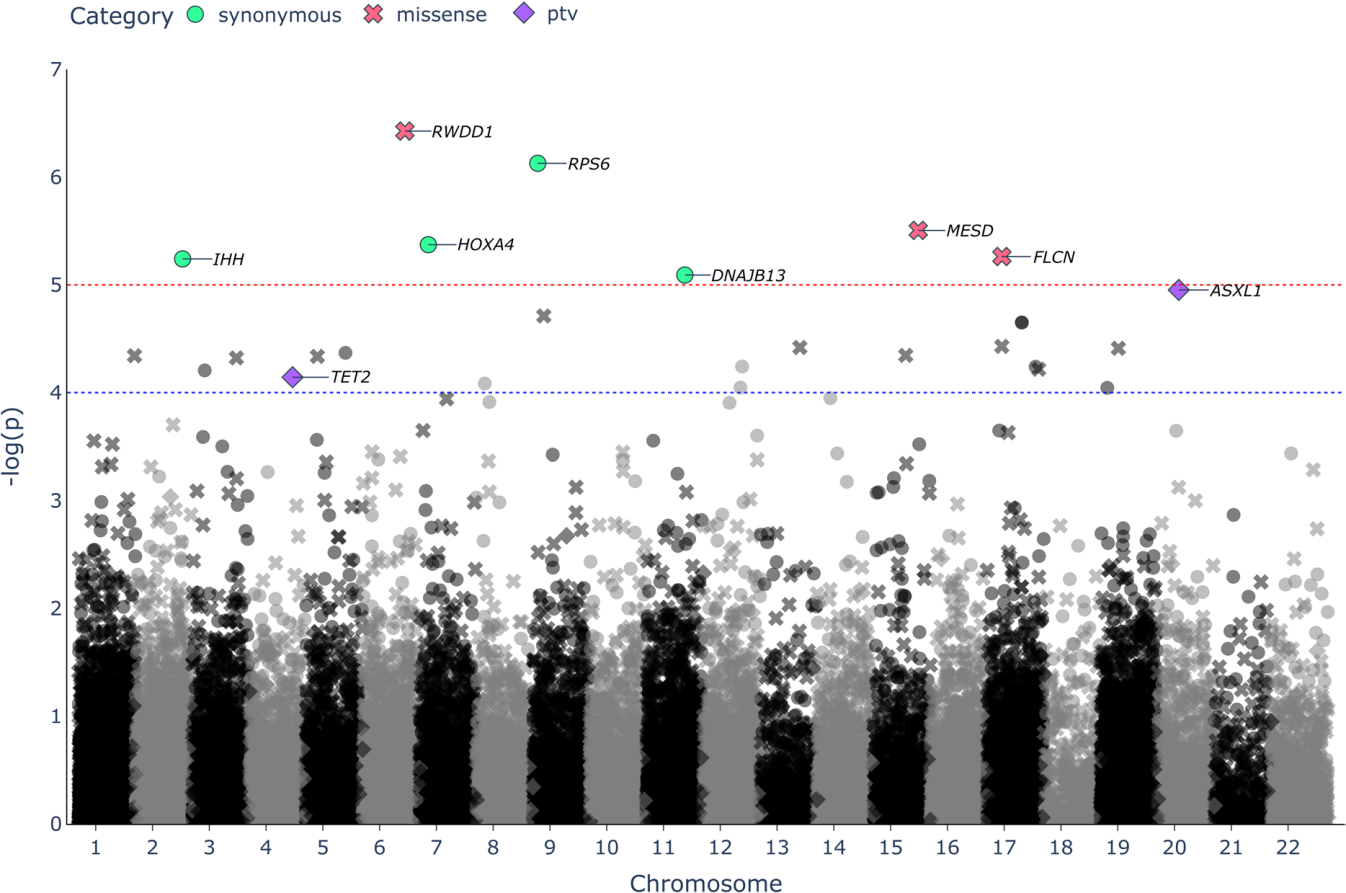
Gene-based association analysis using WES data of 1245 LLI and 4105 younger controls, using rare coding variants (MAF < 0.01) in separate masks for synonymous (circular), missense (x-shaped) and protein-truncating variants (PTV) (diamond-shaped). The significance threshold based on an FDR multiple testing correction of < 0.05 is shown as a red dotted line for synonymous and missense variants and a blue dotted line for PTVs. Genes that passed this threshold for a specific variant mask are labelled with the corresponding gene names and colored. Gene-based test summaries based on synonymous and missense variants were corrected together, whereas test summaries based on PTVs were corrected separately, given that the latter did not employ annotation principal components

Next, we performed a gene-based association analysis using STAAR-O [18, 19] for all protein-coding genes, including all 794,022 rare variants (MAF < 0.01; incl. 532,463 singletons: MAC = 1) (Suppl. Table 1, 2). The gene-based association test STAAR-O is an optimized version of SKAT, burden and ACAT, which aggregate all variants of similar consequence within a gene (i.e. synonymous, missense and PTVs) into one mask to produce a single summary statistic. To increase statistical power, we incorporated a large variety of predictive scores for different categories (see Materials and methods) and used these as variant weights to favor potentially causal variants for the synonymous and missense masks. We identified nine gene-associations that reached exome-wide significance after FDR correction (Fig. 3, Table 2**)**, of which four were based on synonymous variants—*RPS6* (adjusted *p*-value = 0.0142), *HOXA4* (adjusted *p*-value = 0.0365), *IHH* (adjusted *p*-value = 0.0365) and *DNAJB13* (adjusted *p*-value = 0.0442), three on missense variants—*RWDD1* (adjusted *p*-value = 0.0142), *MESD* (adjusted *p*-value = 0.0365) and *FLCN* (adjusted *p*-value = 0.0365), and two on PTVs—*ASXL1* (adjusted *p*-value = 0.0092) and *TET2* (adjusted *p*-value = 0.0297)*.* As with the single-variant analysis, conditioning the analysis on *APOE* genotype did not significantly impact the results of the gene-based analysis. Neither did we observe significant evidence of population structure (Suppl. Figure 1b-d).

**Table 2:** Genes which reach exome-wide significance in gene-based association analysis of WES data from a cohort of 1245 LLI and 4105 younger controls. STAAR-O was used to perform the analysis [18]. *N*, number of variants in the category mask; *cMAC*, cumulative minor allele count

Gene name	Chr	Pos	Category mask	N	cMAC	*P*-value	*P*-value (FDR-adjusted)
*RWDD1*	6	116,571,409	Missense	8	11	3.74 × 10^−07^	0.0142
*RPS6*	9	19,375,715	Synonymous	6	15	7.44 × 10^−07^	0.0142
*MESD*	15	80,946,289	Missense	11	15	3.12 × 10^−06^	0.0365
*HOXA4*	7	27,128,507	Synonymous	11	14	4.24 × 10^−06^	0.0365
*FLCN*	17	17,212,212	Missense	41	113	5.46 × 10^−06^	0.0365
*IHH*	2	219,054,424	Synonymous	15	44	5.74 × 10^−06^	0.0365
*DNAJB13*	11	73,951,026	Synonymous	6	20	8.10 × 10^−06^	0.0442
*ASXL1*	20	32,358,330	PTV	7	14	1.11 × 10^−05^	0.0092
*TET2*	4	105,145,875	PTV	16	16	7.20 × 10^−05^	0.0297

A closer look at the variants within each gene-mask revealed that the associations for *RPS6*, *IHH*, and *FLCN* could primarily be explained by the respective variant previously identified in the single-variant association analysis (Suppl. Table 3). This effect also extended to *MESD*, which was driven by a high CADD score missense variant – rs1398617919 (which was present in 3 LLI and no controls and thus not considered in the single-variant analysis). Removing the single variants from the gene-based analysis resulted in a loss of the association signal for these genes. The association signals that we detected for *DNAJB13* and *HOXA4* were based on several variants within each gene with opposing effects (i.e. different variants with OR below and above 1 in the same gene).

The association of *RWDD1* with longevity, which stood out as the strongest amongst all genes, was based on a cumulative burden effect of eight missense variants (Suppl. Table 4), five of which were singletons. The singletons were present only in LLI, with a sixth variant identified in two LLI and no controls. The remaining two variants were each detected in a single LLI and a single control. The rare alleles were all present in different individuals. The average CADD of the eight missense variants was high at 22.3, with one variant exceeding 30, suggesting that they likely have an effect at the protein level. The variants exhibited balanced VAFs, indicating that they are of germline origin rather than somatic mutations (see below). Given that most alleles were absent in controls, we compared the allele frequencies of *RWDD1* missense variants in LLI to those of NFE. Except for the variant at position 6–116590347(-G-A), which was entirely absent, all variants displayed extremely low allele frequencies in NFE. To assess the likelihood of detecting missense variants in *RWDD1* with a cMAC ≥ 9 in a sample-set with the same size as our LLI group, we employed a binomial test, using the total sum of MAFs of all *RWDD1* missense variants reported for NFE (sum of MAFs = 0.001191). We observed that this likelihood was low at approximately 0.36% (i.e. binomial *p*-value = 0.0036).

We additionally observed a significant burden effect of PTVs in LLI for the genes *ASXL1* and *TET2*, both of which are known drivers of CHIP. CHIP is typically the consequence of somatic mutations in genes like *ASXL1* or *TET2* within haematopoietic stem cells that lead to their clonal expansion [38]. Whilst CHIP and CHIP-driver mutation have been identified in individuals of almost all age groups, there is a strong positive correlation between the occurrence of CHIP and age [39]. This suggests that the PTVs that we identified in *ASXL1* and *TET2* are most likely somatic mutations, which is further supported by the fact that the VAF of most of these PTVs were skewed towards the reference allele (Suppl. Table 5). Our attempts to remove CHIP-driving mutations (see Materials and methods) may therefore not have been strict enough.

### Gene-set over-representation analysis

To gain an overview of potential functional implications of our findings, we performed a gene-set over-representation analysis (ORA) using terms in GO:BP [24] (Table 3) as well as KEGG [25] (Table 4). Here, we combined both our previous strategies, including genes that contained at least one exome-wide significant single variant and genes that reached exome-wide significance in the gene-based analysis (excluding *ASXL1* and *TET2*, as these findings were based on somatic mutations). In total, 11 genes were considered for ORA. We identified a significant over-representation of genes in mTOR (mechanistic target of rapamycin) signaling (based on both enrichGO and enrichKEGG): *FLCN* and *SIK3* positively regulate TOR-Complex 1 (TORC1) and *RPS6* is an important downstream target of TORC1 signaling involved in protein synthesis. Furthermore, we also observed that genes in the pathway “Proteoglycans in cancer” (KEGG), namely *RPS6* and *IHH*, were significantly over-represented.

**Table 3:** Gene-set over-representation analysis using R package clusterProfiler [23] function enrichGO with GO:BP [24] showing pathways with a minimum of 2 matching genes out of 11 tested genes

GO ID	Term	Number of matching genes/Total genes in term	Matching genes	*P*-value	*P*-value adjusted
GO:0031929	TOR signaling	3/152	*RPS6/FLCN/SIK3*	9.04 × 10^−05^	**2.30 × 10**^**−02**^
GO:1,904,263	Positive regulation of TORC1 signaling	2/49	*FLCN/SIK3*	3.91 × 10^−04^	**4.98 × 10**^**−02**^
GO:0032008	Positive regulation of TOR signaling	2/67	*FLCN/SIK3*	7.30 × 10^−04^	6.21 × 10^−02^
GO:1,903,432	Regulation of TORC1 signaling	2/88	*FLCN/SIK3*	1.26 × 10^−03^	7.30 × 10^−02^
GO:0038202	TORC1 signaling	2/94	*FLCN/SIK3*	1.43 × 10^−03^	7.30 × 10^−02^
GO:0048706	Embryonic skeletal system development	2/127	*HOXA4/IHH*	2.59 × 10^−03^	1.00 × 10^−01^
GO:0032006	Regulation of TOR signaling	2/133	*FLCN/SIK3*	2.84 × 10^−03^	1.00 × 10^−01^
GO:0002181	Cytoplasmic translation	2/149	*RWDD1/RPS6*	3.55 × 10^−03^	1.00 × 10^−01^

**Table 4:** Gene-set over-representation analysis using R package clusterProfiler [23] function enrichKEGG [25] showing pathways with a minimum of 2 matching genes out of 11 tested genes

Category	KEGG ID	Pathway	Number of matching genes/total genes in term	Matching genes	*P*-value	*P*-value adjusted
Environmental Information Processing	hsa04150	mTOR signaling pathway	2/153	*RPS6/FLCN*	1.05 × 10^−03^	**7.76 × 10**^−**03**^
Human Diseases	hsa05205	Proteoglycans in cancer	2/196	*RPS6/IHH*	1.73 × 10^−03^	**7.76 × 10**^−**03**^

### Whole pathway analysis

Lastly, to assess whether entire pathways may exhibit an association with longevity, we performed a variant-set analysis for 303 pathways from KEGG (Suppl. Table 6). Similar to the gene-based analysis, the pathway analysis incorporated all variants within all genes of a specific pathway. As may be expected from ORA, “mTOR signaling pathway” was one of the top pathways (ranked 2nd, *p* = 3.4 × 10^−4^, unadjusted ACAT-V – missense variants), though overall, no pathway reached statistical significance after correcting for multiple testing. Again, excluding the previously identified exome-wide significant single variants also removed this association signal. Interestingly, “FoxO signaling pathway”, known to influence longevity, was also amongst the top hits (ranked 11 th, *p* = 8.6 × 10^−3^, unadjusted ACAT-V – synonymous variants). *FOXO3* itself reached nominal significance in the gene-based analysis (*p* = 5.9 × 10^−3^, unadjusted STAAR-O – synonymous variants).

### Supporting evidence

Due to the uniqueness of our study cohort and WES data, there is currently no dataset of comparable size and ancestry available to conduct a full-scale replication of our findings. Nonetheless, we performed a look-up of exome-wide significant single variants as well as any *RWDD1* missense variants in two longevity WES datasets of smaller size and/or distinct ancestry.

The first cohort was comprised of 515 Ashkenazi Jewish (AJ) LLI and 496 matched younger controls [27]. We observed that only three of the seven exome-wide significant variants were present in the dataset, *IHH-rs149554120-T*, *SIK3-rs147730555-G* and *FLCN-rs41419545-T*, likely due to the substantial population-genetic differences between AJ and Europeans [40]. None of the variants showed a nominally significant association, which, given the smaller sample size, was not surprising. However, they did show the same trend as in our data, with centenarians exhibiting an OR > 1 for all three rare alleles (Table 5). We observed no *RWDD1* missense variants at all in either LLI or controls.

**Table 5:** Look-up of single-variant associations and *RWDD1* missense variants in a WES dataset of 515 Ashkenazi Jewish (AJ) centenarians and 496 matched younger controls [27]

Gene	rsID	MAC AJ Centenarian	MAC AJ control	*P*-value	OR
*IHH*	rs149554120	4	2	3.03 × 10^−01^	1.93
*SIK3*	rs147730555	2	1	7.87 × 10^−01^	1.93
*FLCN*	rs41419545	3	0	1.78 × 10^−01^	6.76

The second cohort consisted of 179 Dutch members of the LLS and 98 unrelated younger Dutch controls from BBMRI-NL [29]. We again identified *IHH-rs149554120-T* and *FLCN-rs41419545-T*, which were present in 1 and 2 LLS members, respectively, but not in the controls. We additionally identified the very rare *RWDD1* missense variant, *rs147351925-G*, which was present in a single LLS member (Table 6). This variant was also present in two German LLI from our cohort.

**Table 6:** Look-up of single-variant associations and *RWDD1* missense variants in a WES dataset of 179 members of the Leiden Longevity Study (LLS) and 98 BBMRI controls [29]

Gene	rsID	MAC LLS	MAC BBMRI
*IHH*	rs149554120	1	0
*FLCN*	rs41419545	2	0
*RWDD1*	rs147351925	1	0

Whilst the findings from either cohort do not provide a statistically significant replication, they do offer support for our analysis results.

## Discussion

The aim of this study was to examine the influence of rare coding variants on human longevity by analyzing WES data from 1245 German LLI and 4105 geographically matched younger controls. We discovered significant associations at both the single-variant and gene level, with a strong over-representation of genes involved in mTOR signaling. Three rare variants in mTOR-pathway genes—*RPS6*, *FLCN* and *SIK3*—were notably enriched in LLI. *RWDD1* stood out as a promising longevity candidate due to an increased burden of rare missense variants in LLI. Additional associations were observed with *PRAC2*, *SLC16A6*, *FOCAD*, *IHH*, *MESD*, *HOXA4* and *DNAJB13*. We also detected a significant presence of protein-truncating variants in *ASXL1* and *TET2*, likely as a consequence of CHIP.

In addition, we observed an excess of singletons in LLI compared to younger controls. This discrepancy may potentially be explained by analyzing the past population dynamics in Europe and more specifically in Germany. The explosive population growth in Europe and in Germany over the last centuries resulted in an overabundance of rare alleles and specifically singletons [41]. This was also reflected in our data, where 62.7% of all exonic variants were singletons. However, with the beginning of the twentieth century, fertility rates in Germany started to drop drastically and relative population growth slowed down (https://population.un.org/wpp/). It is conceivable that the difference in singletons between LLI, who were born around the 1900 mark, and younger controls arose due to these changes in relative population growth [42]. Nonetheless, this hypothesis requires more careful investigation.

**Fig. 4:**
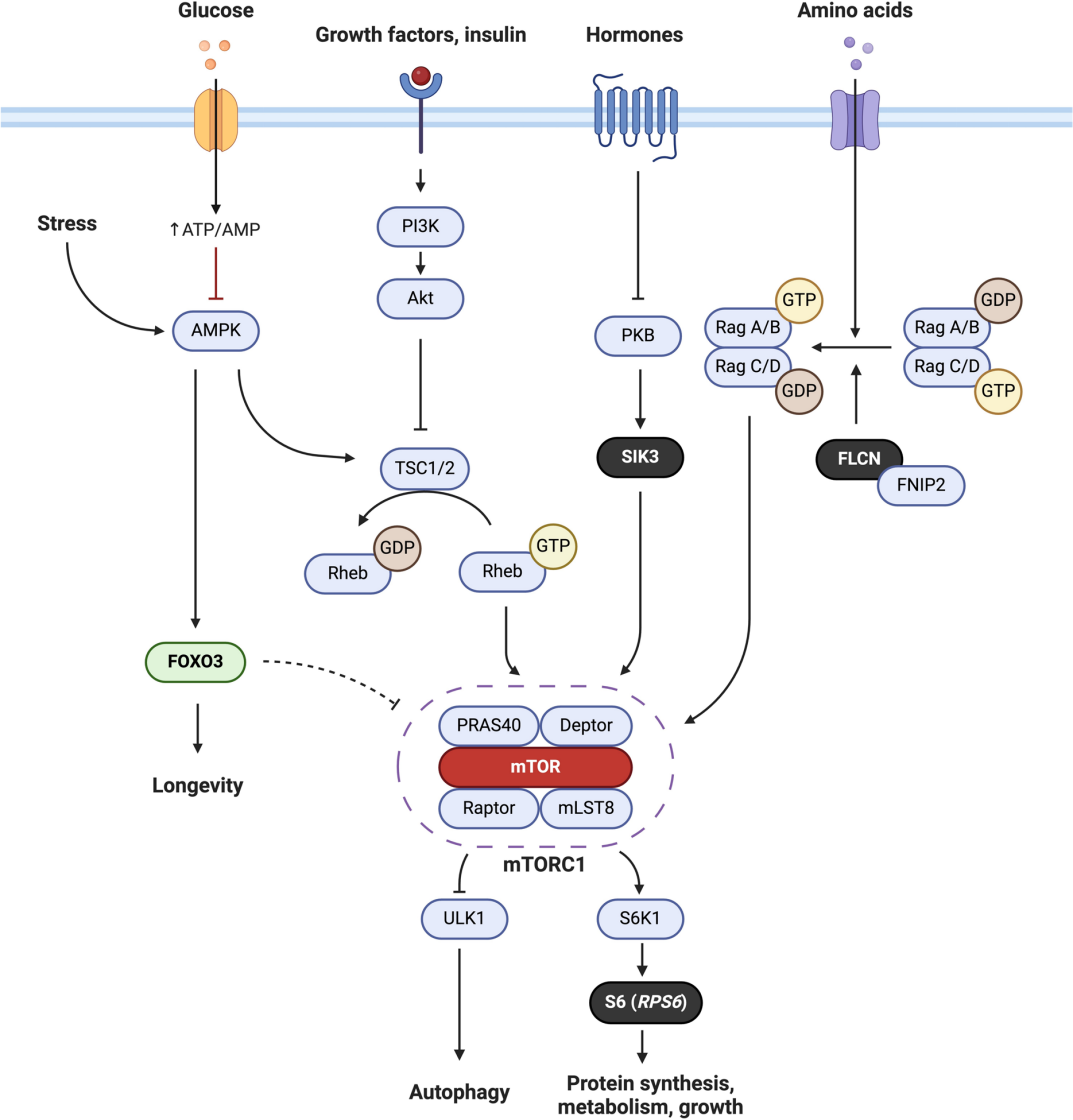
mTORC1 signaling pathway showing genes (or rather their gene products) that reached exome-wide significance in black. The mTOR pathway is part of a highly evolutionarily conserved nutrient sensing network, and an inhibition of this pathway has been shown to promote longevity and lifespan in model organisms. AKT, protein kinase B; AMPK, AMP-activated protein kinase; DEPTOR, DEP domain-containing mTOR-interacting protein; FLCN, folliculin; FNIP2, folliculin interacting protein 2; FOXO3, Forkhead box O3; mLST8, mammalian lethal with SEC13 protein 8; mTOR, mechanistic target of rapamycin; PI3 K, phosphoinositide 3-kinase; PKA, cAMP-dependent protein kinase; PRAS40, proline-rich AKT substrate of 40 kDa; RAGs, Ras-related GTPases; RAPTOR, regulatory-associated protein of mTOR; RHEB, Ras homolog mTORC1 binding; RPS6, ribosomal protein S6; S6 K1, ribosomal S6 kinase 1; SIK3, salt-inducible kinase 3; TSC1/2, tuberous sclerosis complex 1/2; ULK1, Unc- 51-like kinase 1. More information on the mechanisms of how the three potential longevity genes interact with mTOR can be found in prior literature [49, 50, 52]. Created with BioRender

The rare variant association analyses yielded seven novel associations at the variant level and seven at the gene level (excluding CHIP-driver genes), with partial overlap between them. Functionally, we observed that genes involved in mTOR signaling were significantly over-represented amongst our association signals. A previous WES study focussing on rare coding variants in Ashkenazi Jewish centenarians also revealed a significant over-representation of genes related to mTOR signaling, though the specific genes differed [27]. In our study, the association with mTOR signaling was related to three single variants in the genes *RPS6*, *FLCN* and *SIK3* (Fig. 4). mTOR plays a central role in many cellular functions related to growth, proliferation, autophagy, apoptosis and inflammation, within a highly evolutionarily conserved nutrient-sensing network [43]. Studies on model organisms have long established mTOR as a central regulator of aging and longevity [44]. An inhibition of mTORC1, for instance through treatment with rapamycin or mutations in pathway-related genes, induces longevity in many different model organisms [45, 46]. Furthermore, the transcription factor *FOXO3*, which is currently one of the few genes confirmed to influence longevity in humans, functions in the same nutrient-sensing network as mTOR [7, 47, 48]. The gene products of *FLCN* and *SIK3* both positively regulate mTORC1 through distinct upstream signaling cascades [49, 50]. We hypothesize that the protein-altering variants that we identified in each gene, i.e. rs41419545 and, especially, rs147730555 with its high CADD score of 19.45, may result in a reduction of mTORC1 signaling, thereby promoting longevity. In the case of *FLCN*, a prior study already demonstrated how a loss of the *FLCN* product may induce longevity in *Caenorhabditis elegans* [51]. On the other hand, *RPS6* encodes the ribosomal protein S6 (RPS6), a component of the 40S ribosomal subunit and a major downstream target of mTOR directly involved in protein synthesis and cell proliferation [52]. A possible mechanism by which the synonymous variant that we identified in *RPS6* (rs189871470) influences longevity may be through altering the expression of RPS6. Synonymous variants may affect protein expression through different mechanisms such as codon usage bias [53] or an altered mRNA structure [54]. An analysis of rs189871470 using the webtools CodonStatsDB (http://codonstatsdb.unr.edu/) and RNAfold (http://rna.tbi.univie.ac.at/) revealed that (1) the minor allele codon CTT (leucin) has a lower usage compared to the reference CTC codon and (2) there are considerable mRNA structural differences between reference and minor allele coding sequence, which may potentially result in a lower protein expression. Interestingly, a gene expression analysis using whole blood from participants of the LLS showed that *RPS6* expression was lower in the Dutch LLI compared to controls, though the finding was not significant (*p* = 0.055) [55].

Additionally, we identified a potential link between longevity and *RWDD1*, which stood out as the strongest association signal in the gene-based analysis. Unfortunately, there is little research regarding *RWDD1*. According to GTEx, *RWDD1* is expressed across all tissues [56]. The study that first identified *RWDD1* termed its gene product a thymus aging-related protein and showed that the thymic expression of *RWDD1* decreased with age in mice, which may be related to an interplay with the androgen receptor (AR) [57]. According to NCBI, *RWDD1* is predicted to be involved in cellular response to lipids, positive regulation of AR activity and cytoplasmic translation. This information offers functional insights into *RWDD1*’s potential link with longevity, such as the crosstalk between AR and mTOR [58]. We observed that *RWDD1*’s association with longevity was based on an accumulation of missense variants in LLI compared to controls. The fact that the missense variants that we detected had high CADD scores and that based on the NFE reference population missense variants in *RWDD1* are overall less frequent than expected (highest MAF was 0.00032 of variant rs180736980) indicates that the missense variants in *RWDD1* may have a strong effect at the protein level. Whether they ultimately lead to loss- or gain-of-function and how exactly this translates to promoting longevity remains unclear. The findings from the LLS, where we identified one of the exceptionally rare *RWDD1* missense variants in only 179 Dutch LLI, does offer supporting evidence and indicates that the *RWDD1* missense variants may indeed promote longevity.

Other findings encompassed single-variant or gene-based associations involving *PRAC2*, *SLC16A6*, *FOCAD*, *IHH*, *MESD*, *HOXA4*, *DNAJB13*, *ASXL1*, and *TET2.* Interestingly, whilst not explicitly indicated in our over-representation analysis, numerous of these genes have been linked with mTOR signaling. For instance, *IHH* has been shown to promote anti-aging through the downregulation of mTOR signaling in bone marrow-derived mesenchymal stem cells [59]. Both *MESD* and *HOXA4* have been shown to inhibit Wnt/β-catenin signaling, an important regulatory pathway for many cellular functions as well as stem cell renewal and mTORC1 signaling [60–63]. Alongside the mTOR-autophagy axis, *SLC16A6* is a key regulator of the intestinal excretion of ketone bodies that are usually produced during fasting, prolonged physical exertion or a ketogenic diet and have been implicated to promote longevity [64, 65]. On the other hand, *PRAC2*, *FOCAD* and *DNAJB13* have not previously been associated with either mTOR signaling or longevity, and there is overall relatively little research related to these genes. In short, *PRAC2* is specifically expressed in the prostate, bladder, vagina, testis and colon and may be connected to prostate cancer and cellular senescence [66, 67]; *FOCAD* may have an important function in maintaining liver health, with mutations increasing the risk for early-onset liver cirrhosis [68]; *DNAJB13* is primarily expressed in the testis and fallopian tube and has been implied to affect male infertility as well as increasing stress resistance potentially through the antioxidant pathway [69, 70].

As previously mentioned, the gene-associations of *ASXL1* and *TET2*, which were based on PTVs, were most likely the result of CHIP-driver mutations rather than germline variants. We did not identify associations for other prominent CHIP genes, such as *DNMT3A*, *TP53* or *GNB1* [29], likely because we performed a quality control step specifically designed to reduce the number of CHIP-driver mutations in our dataset. The occurrence of these mutations in especially elderly individuals has been observed in numerous studies, and there is overall a strong correlation between age and prevalence of CHIP-driver mutations [29, 39]. Whilst CHIP is associated with an increased risk for cardiovascular disease (CVD) [71] or haematological malignancies [38], a study focussing on the 10-year survival of LLI did not observe a higher mortality for carriers of CHIP-driver mutations [29]. What is more, a recent study postulated that CHIP may exhibit a potential protective effect against Alzheimer’s disease [72]. Regarding PTVs in general, we could not replicate the findings from two prior studies that observed a negative correlation of PTVs and lifespan, neither on an exome-wide level or for specific genes such as *BRCA2* [73, 74].

It is worth noting that especially the single-variant association signals were driven primarily by male LLI and to a lesser extent by female LLI. On the one hand, this may indicate a potential statistical bias, given the much smaller number of male than female LLI in our data. On the other hand, prior studies have indicated that the genetic influence of longevity is likely higher in males [75, 76], which offers another possible explanation for the observed discrepancy. In order to clarify these findings, future longevity cohorts would benefit from prioritizing male LLI during recruitment.

To conclude, this study highlights the contribution of rare variants to the human longevity phenotype. The fact that several variants related to mTOR signaling reached exome-wide significance suggests that the link between this highly evolutionarily conserved pathway and longevity extends from model organisms to humans. Whilst we provided supporting evidence, our findings would benefit from a full-scale replication once an adequate dataset becomes available. Moreover, it would be advantageous for future studies to include larger sample-sets of male LLI, as they are currently underrepresented.

## Supplementary Information

Below is the link to the electronic supplementary material.

ESM 1 (XLSX 2.28 MB)

ESM 2 (PDF 89.9 KB)

ESM 3 (PDF 183 KB)

ESM 4 (TSV 74.8 MB)

## Data Availability

All German samples and information on their corresponding phenotypes were obtained from the PopGen Biobank (Schleswig–Holstein, Germany) and can be accessed through a Material Data Access Form. Information about the Material Data Access Form and how to apply can be found at https://www.uksh.de/p2n/Information+for+Researchers.html.

## Abbreviations

aPC: Annotation principal component
ACAT: Aggregated Cauchy association test
BAM: Binary alignment and map file
BBMRI-NL: Genome of the Netherlands Consortium
CADD: Combined annotation dependent depletion (score)
CHIP: Clonal haematopoiesis of indeterminate potential
cMAC: Cumulative minor allele count
CRAM: Compressed reference-oriented alignment map file
CVD: Cardiovascular disease
FDR: False discovery rate
GDS: Genomic data structure file
GO:BP: Gene Ontology: Biological Processes
GVCF: Genomic variant call format file
KEGG: Kyoto Encyclopedia of Genes and Genomes
LD: Linkage disequilibrium
LLI: Long-lived individuals
LLS: Leiden Longevity Study
MAC: Minor allele count
MAF: Minor allele frequency
mTOR: Mechanistic target of rapamycin
NFE: GnomAD 4.1 reference population non-Finnish Europeans
PTV: Protein-truncating variants
SKAT: Sequence kernel association test
STAAR-O: Optimized variant-set test for association using annotation information
VAF: Variant allele fraction
WES: Whole exome sequencing
