# Supplementary material for: German longevity study reveals novel rare pro-longevity alleles clustering in mTOR signaling pathway
Source: GeroScience. 2025 Apr 15;47(3):4955–71. doi: 10.1007/s11357-025-01640-7 (PMC12181166; doi:10.1007/s11357-025-01640-7)
Supplement: Supplementary file 2 — (PDF 89.9 KB) [file 11357_2025_1640_MOESM2_ESM.pdf]

Supplementary Figure 1

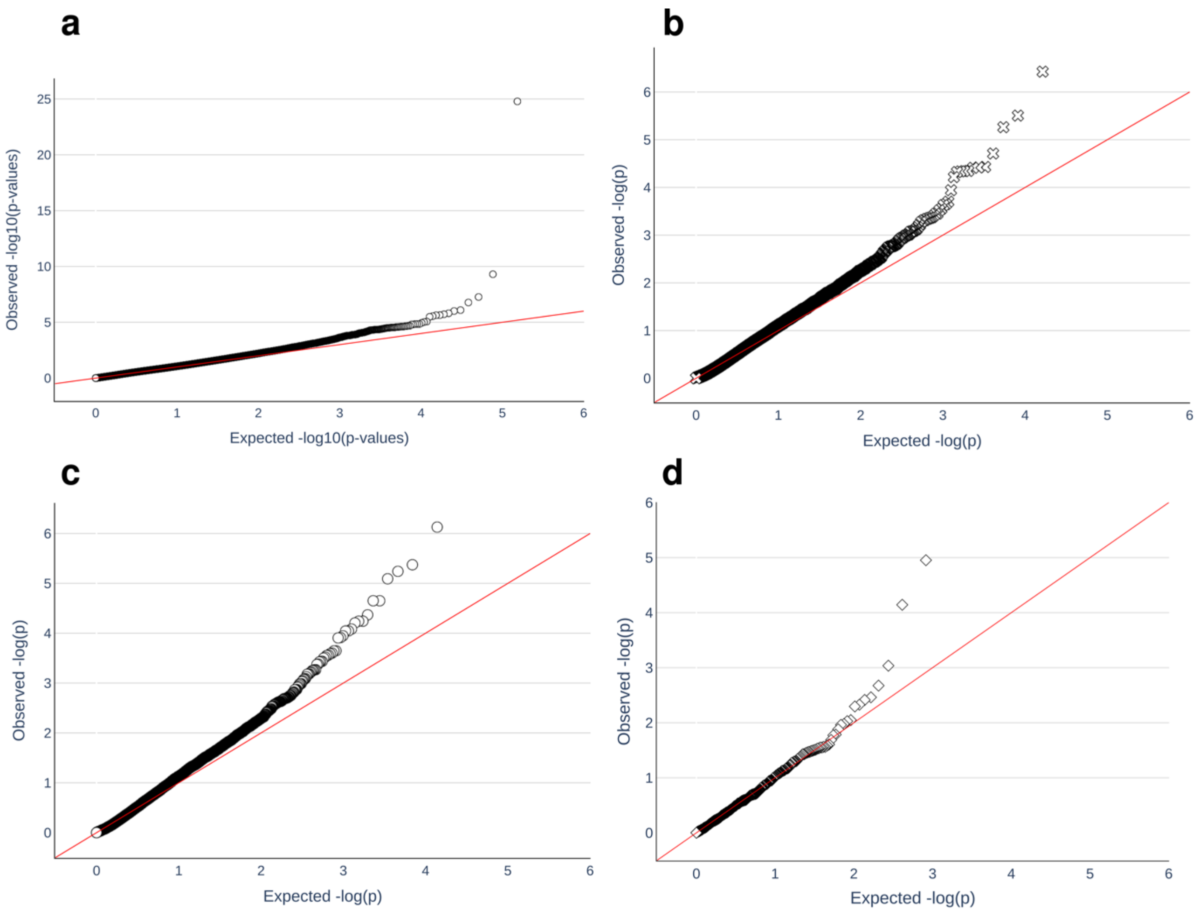

Supplementary Figure 1: QQ-plots for association summary statistics from **a**) single-variant analysis, as well as gene-based analysis for **b)** missense, **c)** synonymous and **d)** protein-truncation variation.
