# Supplementary material for: German longevity study reveals novel rare pro-longevity alleles clustering in mTOR signaling pathway
Source: GeroScience. 2025 Apr 15;47(3):4955–71. doi: 10.1007/s11357-025-01640-7 (PMC12181166; doi:10.1007/s11357-025-01640-7)
Supplement: Supplementary file 3 — (PDF 183 KB) [file 11357_2025_1640_MOESM3_ESM.pdf]

Supplementary File 1

Regeneron Genetics Center

RGC Management & Leadership Team

Aris Baras, Gonçalo Abecasis, Adolfo Ferrando, Giovanni Coppola, Andrew Deubler, Aris Economides, Luca A Lotta, John D Overton, Jeffrey G Reid, Alan Shuldiner, Katherine Siminovitch, Jason Portnoy, Marcus B Jones, Lyndon Mitnaul, Alison Fenney, Jonathan Marchini, Manuel Allen Revez Ferreira, Maya Ghoussaini, Mona Nafde, William Salerno.

Sequencing & Lab Operations

John D Overton, Christina Beechert, Erin Fuller, Laura M Cremona, Eugene Kalyuskin, Hang Du, Caitlin Forsythe, Zhenhua Gu, Kristy Guevara, Michael Lattari, Alexander Lopez, Kia Manoochehri, Prathyusha Challa, Manasi Pradhan, Raymond Reynoso, Ricardo Schiavo, Maria Sotiropoulos Padilla, Chenggu Wang, Sarah E Wolf, Hang Du, Kristy Guevara.

Clinical Informatics

Amelia Averitt, Nilanjana Banerjee, Dadong Li, Sameer Malhotra, Justin Mower, Mudasar Sarwar, Deepika Sharma, Sean Yu, Aaron Zhang, Muhammad Aqeel.

Genome Informatics & Data Engineering

Jeffrey G Reid, Mona Nafde, Manan Goyal, George Mitra, Sanjay Sreeram, Rouel Lanche, Vrushali Mahajan, Sai Lakshmi Vasireddy, Gisu Eom, Krishna Pawan Punuru, Sujit Gokhale, Benjamin Sultan, Pooja Mule, Eliot Austin, Xiaodong Bai, Lance Zhang, Sean O'Keeffe, Razvan Panea, Evan Edelstein, Ayesha Rasool, William Salerno, Evan K Maxwell, Boris Boutkov, Alexander Gorovits, Ju Guan, Lukas Habegger, Alicia Hawes, Olga Krasheninina, Samantha Zarate, Adam J Mansfield, Lukas Habegger.

Analytical Genetics and Data Science

Gonçalo Abecasis, Manuel Allen Revez Ferreira, Joshua Backman, Kathy Burch, Adrian Campos, Liron Ganel, Sheila Gaynor, Benjamin Geraghty, Arkopravo Ghosh, Salvador Romero Martinez, Christopher Gillies, Lauren Gurski, Joseph Herman, Eric Jorgenson, Tyler Joseph, Michael Kessler, Jack Kosmicki, Adam Locke, Priyanka Nakka, Jonathan Marchini, Karl Landheer, Olivier Delaneau, Maya Ghoussaini, Anthony Marcketta, Joelle Mbatchou, Arden Moscati, Aditeya Pandey, Anita Pandit, Jonathan Ross, Carlo Sidore, Eli Stahl, Timothy Thornton, Sailaja Vedantam, Rujin Wang, Kuan-Han Wu, Bin Ye, Blair Zhang, Andrey Ziyatdinov, Yuxin Zou, Jingning Zhang, Kyoko Watanabe, Mira Tang, Frank Wendt, Suganthi Balasubramanian, Suying Bao, Kathie Sun, Chuanyi Zhang.

Therapeutic Area Genetics

Adolfo Ferrando, Giovanni Coppola, Luca A Lotta, Alan Shuldiner, Katherine Siminovitch, Brian Hobbs, Jon Silver, William Palmer, Rita Guerreiro, Amit Joshi, Antoine Baldassari, Cristen Willer, Sarah Graham, Ernst Mayerhofer, Erola Pairo Castineira, Mary Haas, Niek Verweij, George Hindy, Jonas Bovijn, Tanima De, Parsa Akbari, Luanluan Sun, Olukayode Sosina, Arthur Gilly, Peter Dornbos, Juan Rodriguez-Flores, Moeen Riaz, Manav Kapoor, Gannie Tzoneva, Momodou W Jallow, Anna Alkelai, Ariane Ayer, Veera Rajagopal, Sahar Gelfman, Vijay Kumar,

Jacqueline Otto, Neelroop Parikshak, Aysegul Guvenek, Jose Bras, Silvia Alvarez, Jessie Brown, Jing He, Hossein Khiabanian, Joana Revez, Kimberly Skead, Valentina Zavala, Jae Soon Sul, Lei Chen, Sam Choi, Amy Damask, Nan Lin, Charles Paulding.

Research Program Management & Strategic Initiatives
Marcus B Jones, Esteban Chen, Michelle G LeBlanc, Jason Mighty, Jennifer Rico-Varela, Nirupama Nishtala, Nadia Rana, Jaimee Hernandez.

Senior Partnerships & Business Operations
Alison Fenney, Randi Schwartz, Jody Hankins, Anna Han, Samuel Hart.

Business Operations & Administrative Coordinators
Ann Perez-Beals, Gina Solari, Johannie Rivera-Picart, Michelle Pagan, Sunilbe Siceron.
